# Conjoined Twins in Guinea Pigs (*Cavia porcellus*): Case Report

**DOI:** 10.3390/ani16203291

**Published:** 2026-10-19

**Authors:** Medali Cueva-Rodríguez, Jhenny Sánchez-Estela, Jorge Portal-Torres, Giussepe Reyna-Cotrina, Flor L. Mejía, Bessy Castillo-Santamaria, Judith Estela-Manrique, William Bardales Escalante

**Affiliations:** 1 Veterinary Pathology Laboratory, Faculty of Veterinary Sciences, National University of Cajamarca, Cajamarca, Av. Atahualpa, Km. 3, Cajamarca 06003, Peru; jsancheze23_1@unc.edu.pe (J.S.-E.); jportal@unc.edu.pe (J.P.-T.); greynac@unc.edu.pe (G.R.-C.); 2 Agrostology Laboratory, Institute for Livestock and Biotechnology Research, Faculty of Animal Science, Biotechnology, Agribusiness and Data Science, Toribio Rodríguez de Mendoza National University of Amazonas, Chachapoyas 01001, Peru; flor.mejia@untrm.edu.pe; 3 Chemistry Laboratory, Faculty of Agricultural Sciences, National University of Cañete, San Vicente de Cañete 15701, Peru; bcastillo@undc.edu.pe; 4 National Institute of Agricultural Innovation, Baños del Inca Agricultural Research Station, Cajamarca, Jirón Wiracocha, No. S/N, Los Baños del Inca 06004, Peru; jestela@inia.gob.pe; 5 Institute for Research in Livestock and Biotechnology, Faculty of Animal Science, Biotechnology, Agribusiness and Data Science, Toribio Rodríguez de Mendoza National University of Amazonas, Chachapoyas 01001, Peru

**Keywords:** cephalopagus, congenital malformation, guinea pig, omphalopagus, teratology, thoracopagus

## Abstract

This study reports a case of conjoined twins in guinea pigs (*Cavia porcellus*) from the Peru breed, in the Cajamarca region, Peru. A one-year-old female gave birth to normal offspring and a pair of conjoined twins born dead; The latter were carried out with morphological, radiographic and echographic evaluations, and the blood count and serum biochemical profile of the dam were analyzed. The twins presented cephalothoraco–omphalopagus fusion, characterized by a single skull with cyclopia, arhinia and astomy, ventral thoracoabdominal fusion, the pelvis separated and four extremities (four pairs) completely dislocated. Radiographic and ultrasound evaluations identified two hearts and two pairs of kidneys, with preserved gross sonographic morphology and no evident major structural abnormalities. The maternal blood count showed mild hypochromic macrocytosis and mild thrombocytopenia, with the rest of the hematological and biochemical parameters within normal ranges. It was concluded that the hallmarks are compatible with an early developmental disturbance affecting midline structures, although the precise timing and etiology could not be definitively established, which selectively affected the structures of the medial line. This report expands the scarcity of evidence available on serious congenital malformations in guinea pigs.

## Simple Summary

Conjoined twins are a somewhat common malformation in children born physically united. In smaller production animals such as the guinea pig, these cases are extremely rare and few have been scientifically documented. The objective was to describe a case of conjoined twins in children born in the Cajamarca region, Peru. The dam gave birth to three children: both normal and alive, and a pair of conjoined twins who were born dead. The twins share a single head with a single central eye, without a nose or mouth, and are united from the head up to the shoulder, even with their extremities completely uncurled. Radiographic and ultrasonographic examinations identified two hearts and two pairs of kidneys without evident major structural abnormalities in the structures that could be evaluated. This case documents an unusual condition in guinea pigs and provides information on significant congenital malformations in this species in Peru.

## 1. Introduction

In the development of monozygotic twins, conjoined twins represent an unusual genetic malformation, present in both humans and animals of different species, including mammals, birds, reptiles, amphibians, and fish [1]. Twin pregnancy occurs through the fertilization of two separate ova (dizygotic) or through a single fertilized ovum that divides into two genetically identical embryos (monozygotic) [2]. Conjoined twins are monozygotic and monochorionic individuals, joined by a common anatomical part, vascularly connected to each other, who may share one or more organs, and who are of the same sex and genetically identical [3]. The prevalence in human medicine varies from 1 in 50,000 to 1 in 100,000 births, depending on the geographic region [4]; furthermore, they occur more frequently in females than in males, in a ratio of 3:1 [4].

In the veterinary field, conjoined twins have been reported in livestock such as cattle, sheep, and pigs, while in smaller species, reports are infrequent, as occurs in dogs [5,6]. In rodents, reports of this malformation are even rarer, especially in guinea pigs; a few cases of conjoined twins have been detected in this species [7].

Conjoined twins consist of two individuals connected or fused to varying degrees and in different body regions. They can be symmetrical or asymmetrical; in the latter, the fully formed individual is called an authosite, while the other, rudimentary one, is called a parasite [8]. The classification of conjoined twins is based on the anatomical site of fusion, followed by the suffix “pagus,” a Greek term meaning fixation [2]. The main types are omphalopagus (abdomen), thoracopagus (thorax), cephalopagus (upper part of the head to the umbilicus), ischiopagus (pelvis), parapagus (lateral union), craniopagus (head), pygopagus (sacrum), and rachipagus (vertebral column) [9]. Each type has its own embryological basis, which determines particular anatomical structures [10].

Currently, conjoined twins are commonly believed to result from partial fission or secondary fusion [11]. Embryologically, normal twins result from complete separation of the first blastomeres, followed by partial fusion. In conjoined twins, however, it is assumed that incomplete fission of the first two blastomeres occurs, with duplication only of the parts that did separate, suggesting incomplete separation of the embryonic disc during gastrulation, toward the end of the second week of development [8]. The fusion theory, on the other hand, proposes that conjoined twins result from two initially separate monozygotic embryos that fused secondarily and homologously; this is currently the most widely accepted theory [12]. The causes of this anomalous event remain unknown, and no specific genetic or environmental risk factors have been identified [11].

In Peru, the raising of guinea pigs (*Cavia porcellus*), a species native to the Andes, constitutes a means of economic and food development for rural areas, as they are a source of high biological value protein and income through the sale of meat and breeding stock [13]. With an estimated population of 17,380,175 guinea pigs, Peru is considered the leading producer of the species; the largest proportions are concentrated in Cajamarca (19.0%), Cusco (13.5%), Ancash (12.9%), Apurimac (8.0%), and Junin (7.6%) [14]. Despite the productive importance of the species, knowledge about congenital body malformations in guinea pigs is scarce, and to date, only a few cases have been reported in the country and the rest of the world.

Due to the scarcity of reports on this type of malformation in Peru, especially in the Cajamarca region, the present study aimed to describe a case of cephalothoraco–omphalopagus conjoined twins in guinea pigs of the Peru breed, through morphological, radiographic, ultrasound and maternal hematological characterization.

## 2. Materials and Methods

A case of conjoined twins born to an adult female guinea pig (*Cavia porcellus*) of the Peru breed, approximately one year old and weighing 1832 g live weight, was recorded in a family shed in the hamlet of Machaypungo Alto, Bambamarca district, Hualgayoc province, Cajamarca region, Peru. A male of the same breed and age mated with the dam. According to the owner, both animals originated from the same breeding group; therefore, consanguinity was suspected based on the breeding history. However, no formal pedigree records were available to confirm their degree of relatedness, as is commonly the case in family-based guinea pig production systems in the region.

The animals were raised under a family-based rearing system typical of Cajamarca; consequently, data regarding environmental parameters such as temperature or relative humidity are unavailable. The guinea pigs were housed in groups of five guinea pigs in cages made of wood and wire mesh, with straw bedding and natural ventilation. The cages were cleaned approximately once a week. Their diet consisted mainly of oats, alfalfa, and clover. During the summer, their diet was supplemented with ground corn and commercial guinea pig concentrate purchased in bulk; consequently, no information was available regarding the manufacturer, brand, or nutritional composition. Water was provided ad libitum only during periods of higher ambient temperature.

The gestation proceeded without apparent complications. The birth was spontaneous and did not require assistance. The litter consisted of four pups: two live (one male and one female) and two conjoined pups that were stillborn. The healthy pups were separated to continue nursing without complications. The dam remained in normal clinical condition after giving birth and exhibited appropriate maternal behaviour toward the healthy pups.

Radiographic studies were performed using a clinical X-ray unit (TXR 325, Tingle X-Ray Products, Vance, AL, USA) and a flat panel X-ray detector (MSL-FP06, Medsinglong Global Group Co., Ltd., Guangzhou, China). Dorsoventral and ventrodorsal projections were chosen to evaluate the skeletal structure of the fused individuals. An ultrasound study was also performed using a portable veterinary scanner (SonoEye P1, CHISON Medical Technologies Co., Ltd., Wuxi, China) equipped with a linear transducer operating at 9.0 MHz to evaluate the internal organs of the thoracic and abdominal cavities. The specific model of the transducer was not recorded. Subsequently, an external morphological examination was conducted using a flexible measuring tape. The recorded variables included head length and width, neck circumference, abdominal girth, hip girth, total animal length, body length, height at the withers, forelimb and hindlimb length, and cannon bone girth.

Additionally, a maternal blood sample was obtained by venipuncture of the lateral saphenous vein of the hind limb. For complete blood count, 2 mL of whole blood was collected into a tube with EDTA as anticoagulant and analyzed using an automated hematology analyzer (BC-3600, Mindray, Shenzhen, China). An additional 2 mL blood sample was collected into a tube without anticoagulant for serum biochemical analysis, using QCA liquid chemistry reagents (Química Clínica Aplicada S.A., Amposta, Spain) and a split-beam UV/Vis spectrophotometer (Zuzi 4510/4, Auxilab S.L., Beriáin, Spain). These analyses were carried out with the aim of analyzing possible maternal metabolic alterations potentially associated with the occurrence of the congenital malformation.

## 3. Results

### 3.1. External Morphological Findings

**Figure 1:**
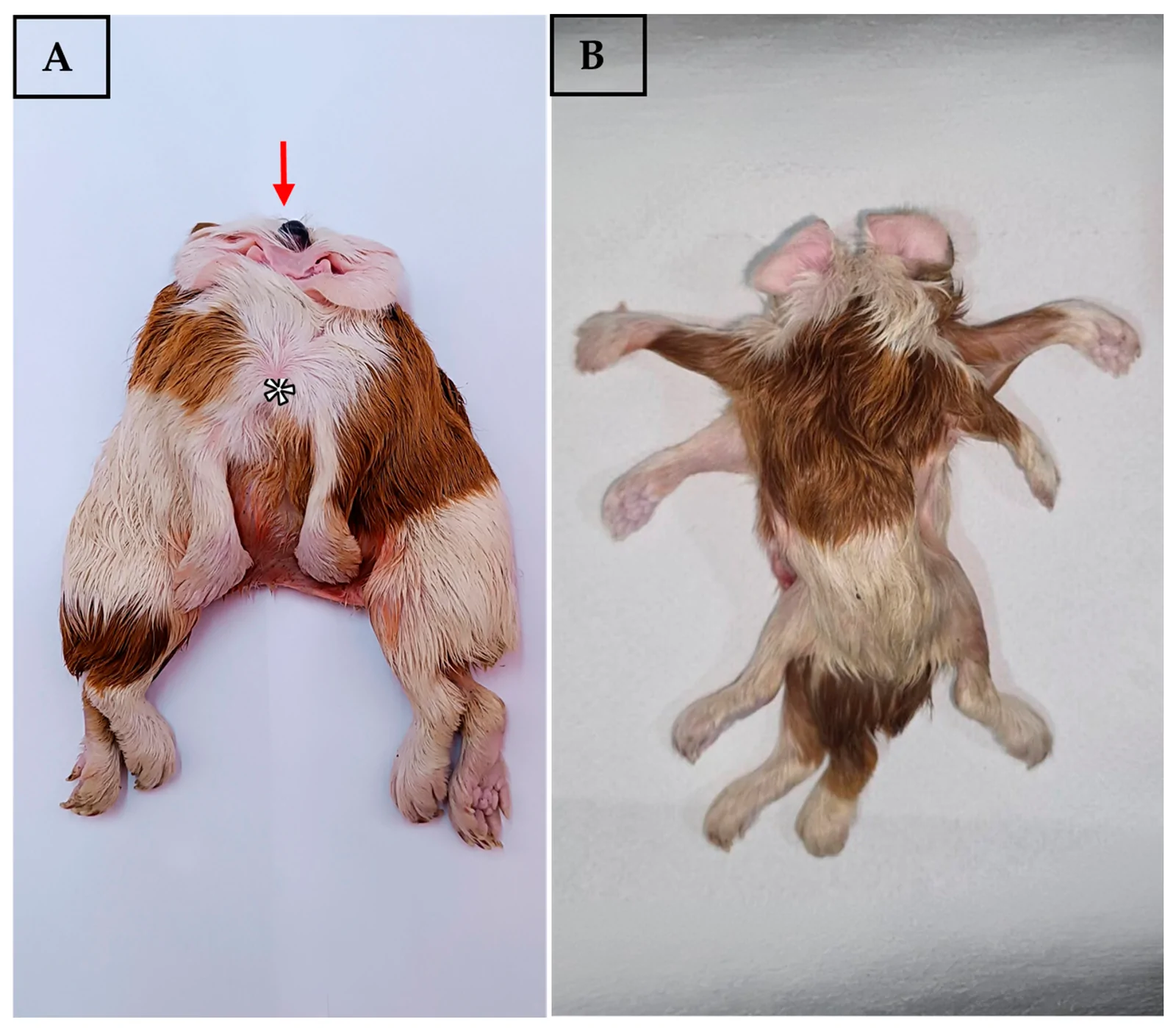
Morphological characteristics of conjoined offspring with cephalothoraco–omphalopagus fusion in guinea pig (*Cavia porcellus*). (**A**) Ventral view showing the single head with cyclopia (arrow), absence of nose and mouth, thoracoabdominal fusion (asterisk) and separation of the bodies at the inguinal level; (**B**) Dorsal view showing eight total limbs (four per individual) with normal development.

The conjoined twins were stillborn. Based on the evaluation of the external genitalia, both were male. Their combined weight was 172 g. The twins presented characteristics consistent with a cephalothoraco–omphalopagus configuration, with cranial and ventral fusion from the thoracic region to the umbilical level, and a parallel and symmetrical cephalocaudal orientation (Figure 1A). Two bodies joined at the cranial level were observed, sharing a single head with severe craniofacial malformations: complete absence of a nose, absence of a mouth opening, and the presence of a single eyeball in a central position, consistent with cyclopia (Figure 1A). Four symmetrically arranged ear pinnae were identified on the dorsolateral region of the skull (Figure 1A,B).

The anterior thoracic wall showed complete fusion, with a shared sternum and common ventral rib cage (Figure 1A). Four thoracic limbs (two pairs) were identified with apparently complete development of bony structures, joints, and digits (Figure 1B). External inspection did not allow for determining the degree of sharing or duplication of intrathoracic organs. Likewise, the ventral abdominal wall showed complete fusion from the xiphoid process to the umbilical region. Superficial palpation suggested the presence of shared abdominal viscera, particularly an expanded liver and intertwined intestinal loops (Figure 1A). The exact degree of sharing of individual abdominal organs could not be determined due to the absence of anatomical dissection.

Two completely separate and anatomically distinct pelvises were evident. Four hind limbs (two pairs) were identified with complete development of bony structures, joints, and digits (Figure 1B). Each twin presented individual perineal structures, suggesting a separation of the distal gastrointestinal and urogenital tracts (Figure 1B).

Comparative morphological analysis between the two twins revealed moderate morphometric differences between the individuals, indicating relatively symmetrical development despite the congenital fusion (Table 1). Twin 2 showed greater dimensions in body length and limbs, while perimeter measurements were comparable between the two (Table 1). The inability to obtain individual measurements of head, neck, and thoracic perimeter reflected the nature of the cephalothoraco–omphalopagus fusion.

### 3.2. Radiographic Findings

**Figure 2:**
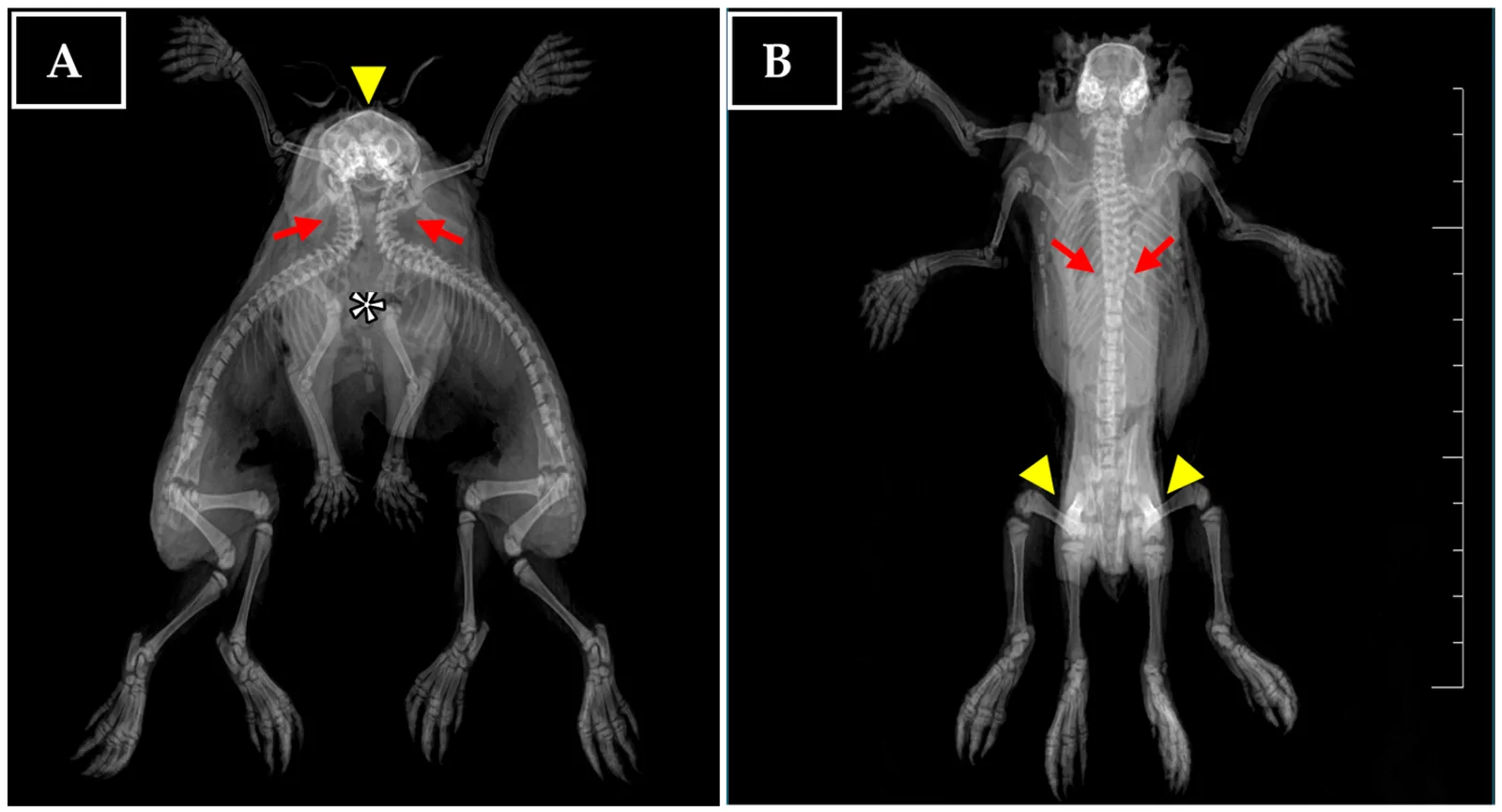
Full-body radiographs of cephalothoraco–omphalopagus conjoined twins in guinea pigs. (**A**) Dorsoventral projection showing a single malformed skull (arrowhead) with severe craniofacial malformation and cyclopia, absence of nasal bones, two converging vertebral columns with cervical–thoracic fusion (arrows), four shoulder girdles, ventral thoracoabdominal fusion with soft tissue overlap (asterisk), two separate pelvises, and flexed limbs. (**B**) Ventrodorsal projection, evidencing axial duplication with two vertebral columns (arrows), four shoulder girdles (arrowhead), two independent pelvic girdles, four fully developed thoracic and four pelvic limbs with lateral extension.

The classification of cephalothoraco–omphalopagus conjoined twins was confirmed, characterized by fusion from the cranial to the umbilical region. Two complete vertebral columns were observed, with cranial cervical–thoracic fusion and lumbar separation, evidencing incomplete axial duplication (Figure 2A). Four completely separate pectoral girdles and two pelvic girdles were identified, along with normal development of all eight limbs (Figure 2B). These findings indicated that the malformation primarily affected the axial skeleton and midline structures, while preserving the development of the appendicular skeleton. No other significant skeletal anomalies were identified on radiographic examination.

### 3.3. Ultrasound Evaluation

**Figure 3:**
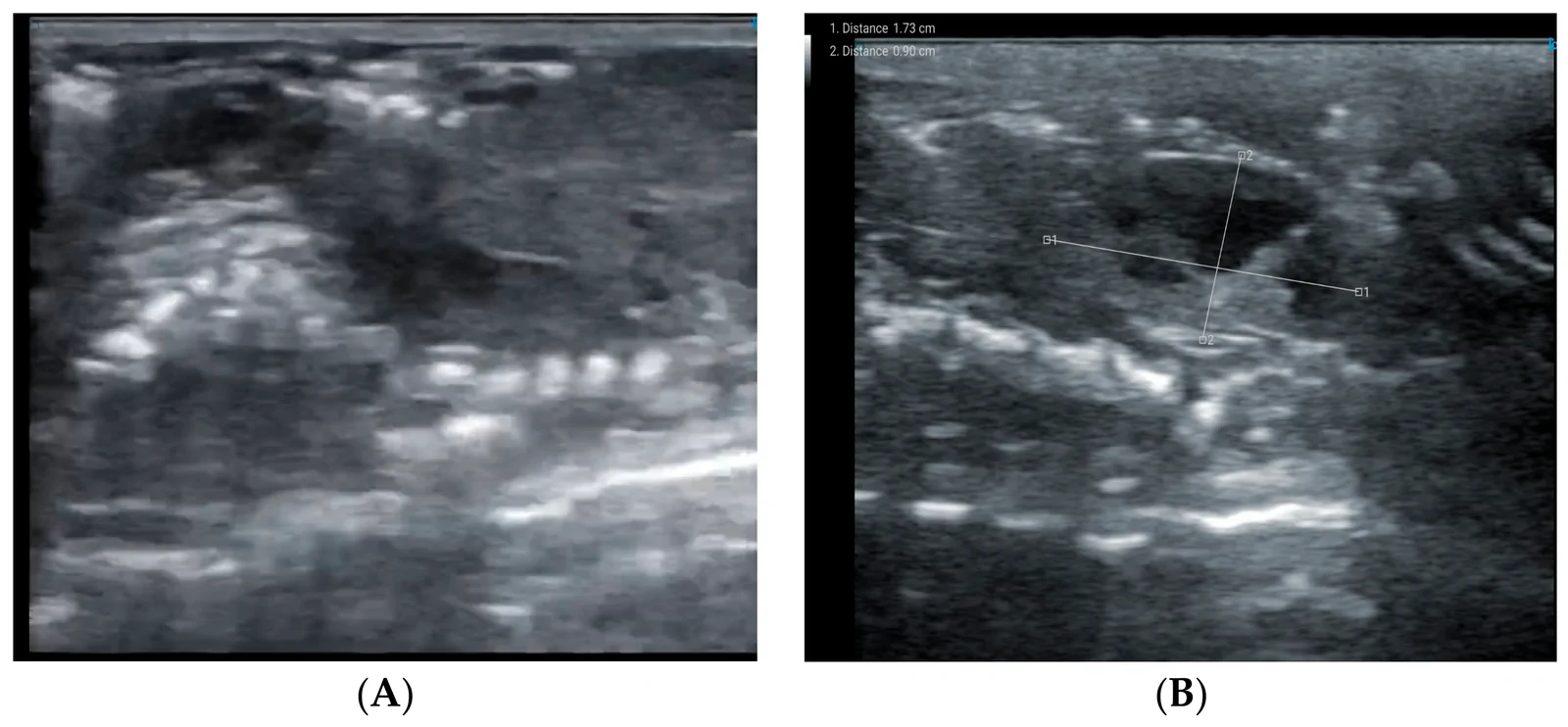
Renal ultrasound evaluation of the left twin in cephalothoraco–omphalopagus conjoined guinea pigs. (**A**) Left kidney in longitudinal plane showing preserved reniform morphology, adequate corticomedullary differentiation, cortex of homogeneous echogenicity and a well-defined hyperechoic renal sinus, without evidence of pelvic dilation or focal lesions. (**B**) Right kidney in longitudinal plane, with an approximate length of 1.73 cm and a transverse diameter of 0.90 cm, preserved reniform morphology, parenchyma of homogeneous echogenicity, adequate corticomedullary differentiation and a hyperechoic renal sinus, without structural alterations.

**Figure 4:**
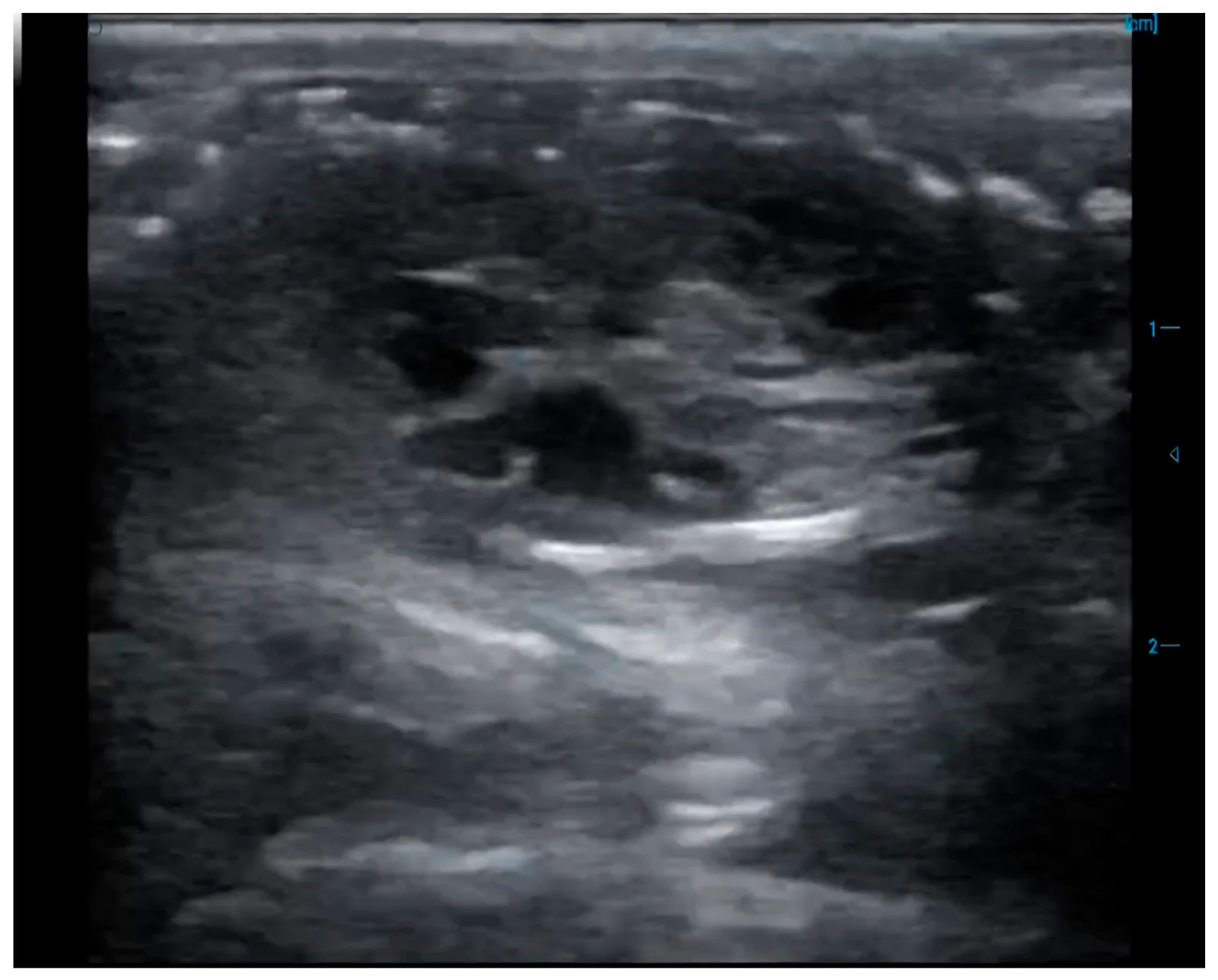
Bilateral renal ultrasound evaluation of the right twin in cephalothoraco–omphalopagus conjoined guinea pigs. Both kidneys present preserved reniform morphology, defined contours, parenchyma of homogeneous echogenicity, adequate corticomedullary differentiation, hyperechoic renal sinus, without evidence of pelvic dilation or focal lesions.

**Figure 5:**
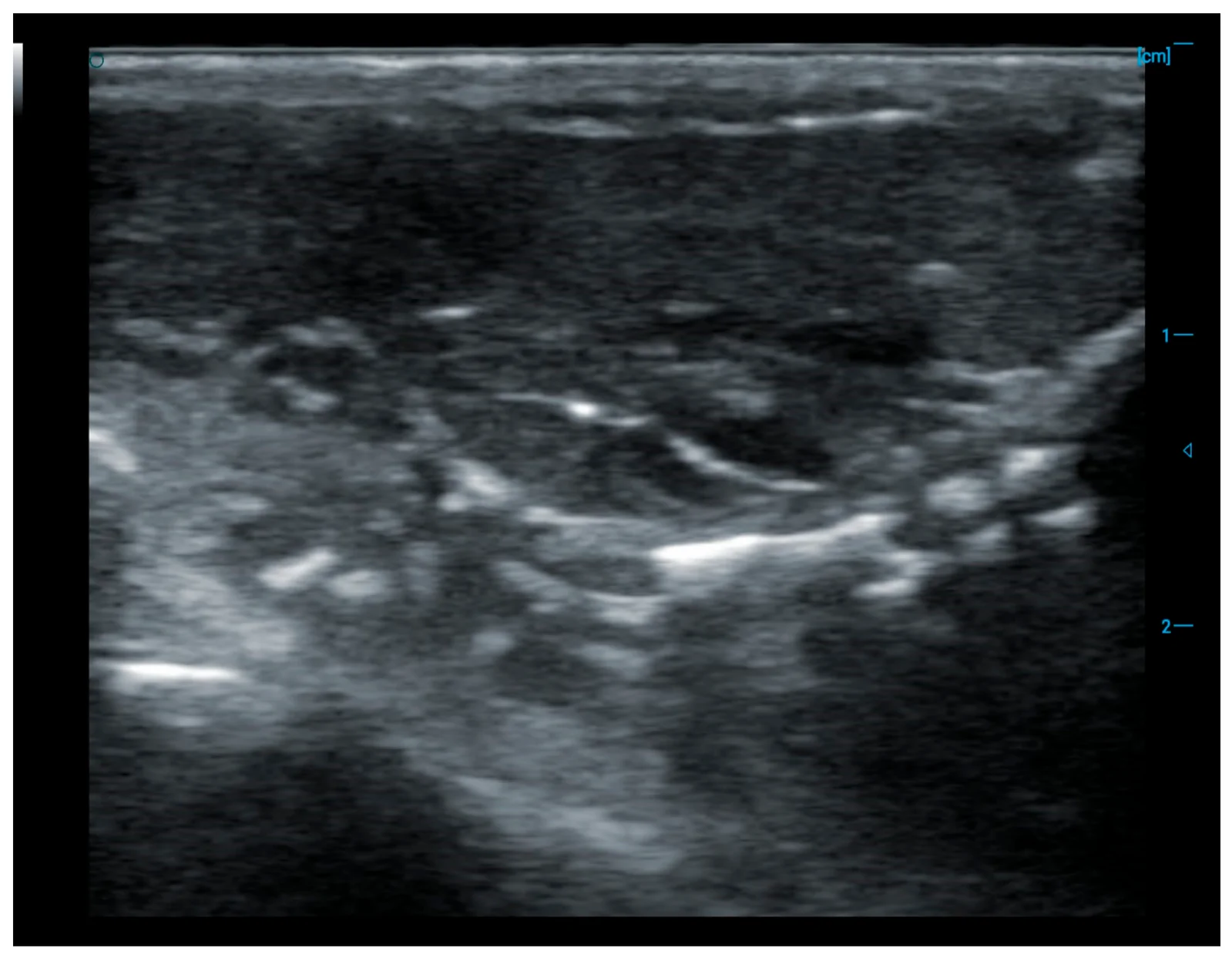
Cardiac ultrasound evaluation of the right twin in cephalothoraco–omphalopagus conjoined guinea pigs. Longitudinal plane showing preserved cardiac silhouette, visible cavities, homogeneous myocardial echogenicity and absence of pericardial effusion or evident structural alterations.

**Figure 6:**
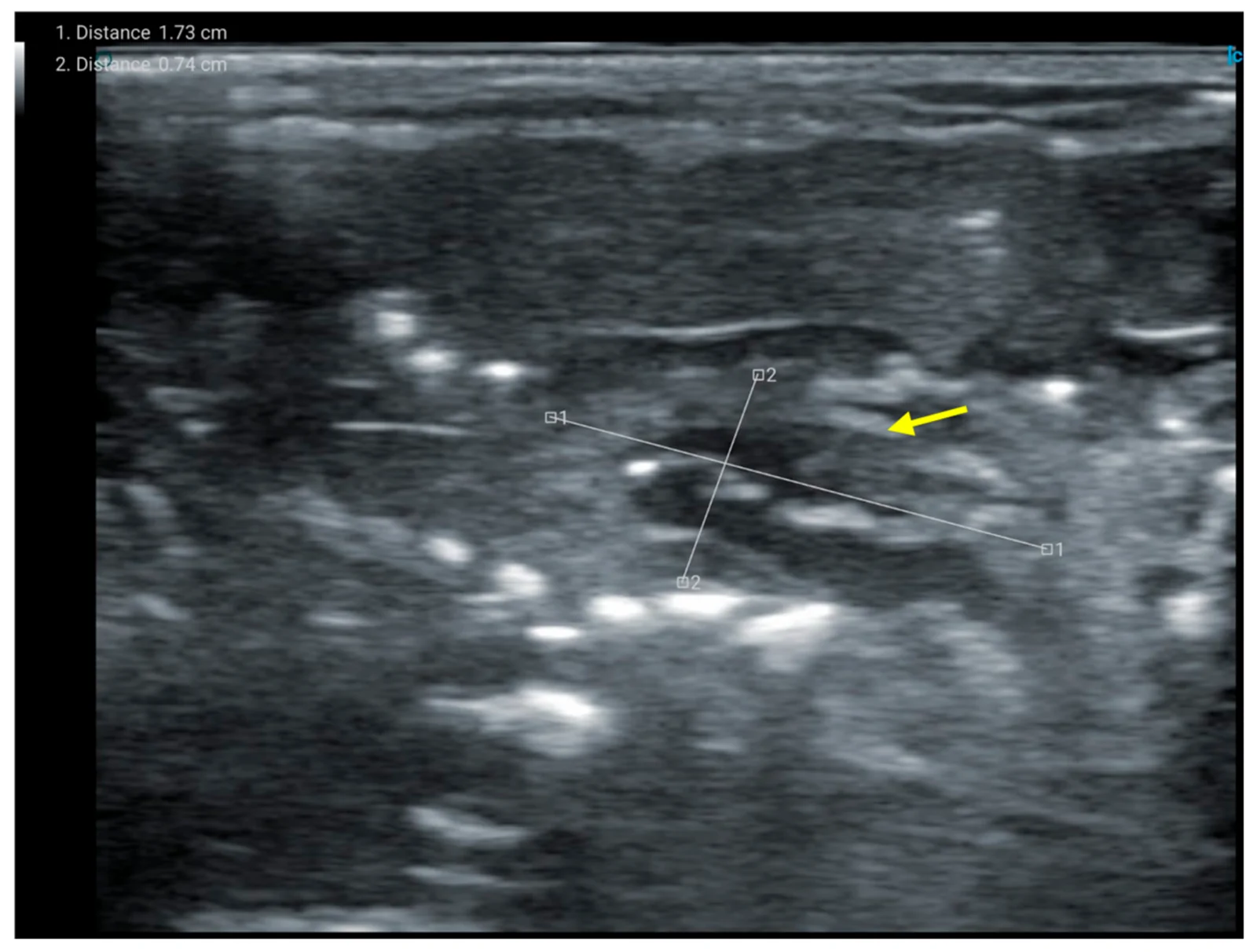
Thoracic ultrasound evaluation of the left twin in cephalothoraco–omphalopagus conjoined guinea pigs. Longitudinal plane showing echogenic lung fields in the anterior region, and a heart with preserved silhouette in the posterior region (arrow), with defined contours and normal anatomical relationship.

The bilateral kidneys presented adequate dimensions for neonates, preserved kidney-shaped morphology, and appropriate corticomedullary differentiation, without evidence of structural abnormalities (Figure 3 and Figure 4). The hearts of both twins showed a preserved cardiac silhouette, with recognizable chambers and homogeneous myocardial echogenicity, without pericardial effusion or major structural alterations (Figure 5 and Figure 6).

**Figure 7:**
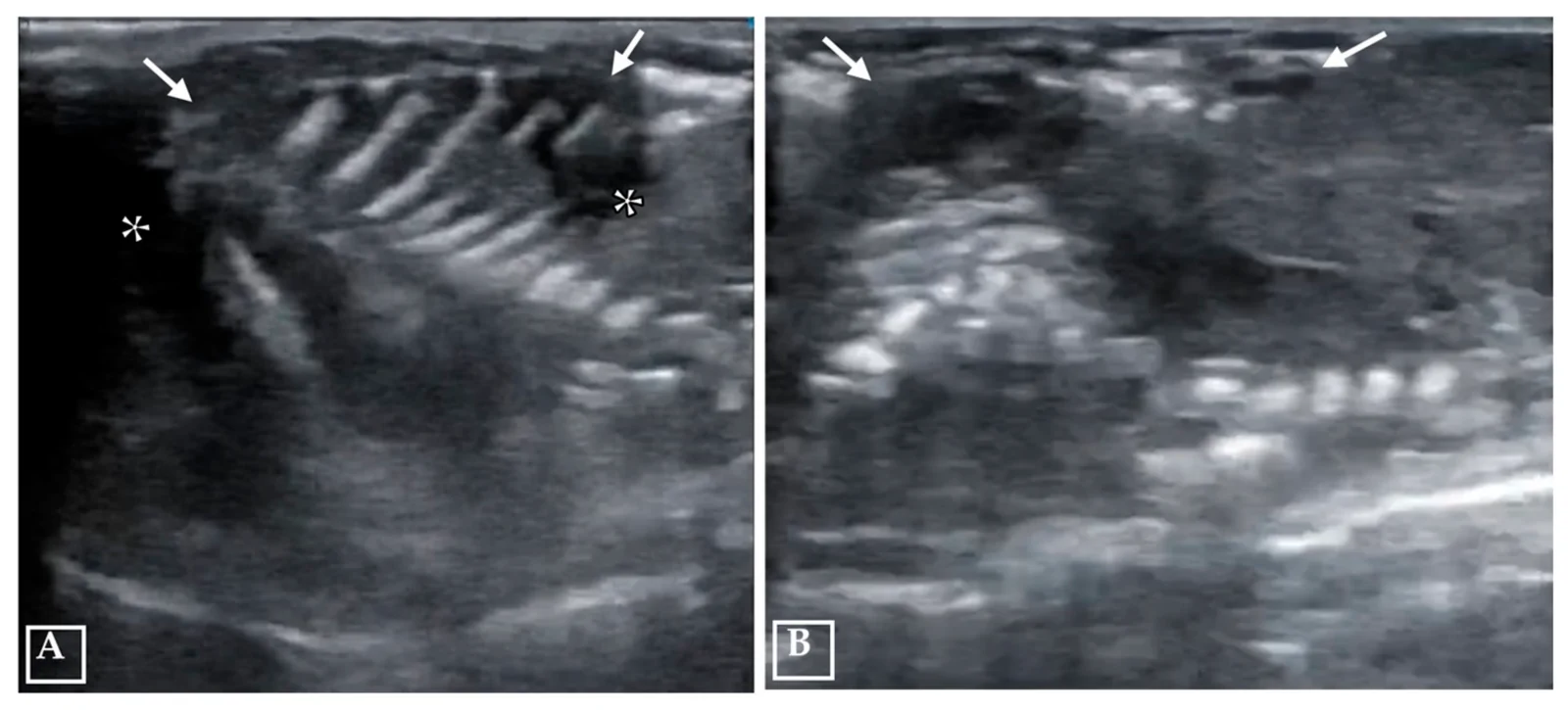
Thoracic ultrasound evaluation of conjoined twins in cephalothoraco–omphalopagus guinea pigs. Marked thoracic edema is observed, evidenced by diffuse thickening of the soft tissues (arrows), heterogeneous increase in echogenicity, and the presence of irregular hypoechoic areas consistent with interstitial fluid accumulation (asterisks), with partial loss of normal anatomical definition. (**A**) Thoracic wall of the right twin showing severe subcutaneous edema. (**B**) Thoracic wall of the left twin with similar findings.

The lung fields presented marked diffuse hyperechogenicity. Severe thoracic soft tissue edema was observed in both twins, evidenced by diffuse thickening of the subcutaneous tissue and multiple irregular hypoechoic areas consistent with interstitial fluid accumulation (Figure 7). These ultrasound findings indicated that, despite severe craniofacial malformations and thoracoabdominal fusion, the evaluated internal organs showed no major structural abnormalities on ultrasonographic examination. However, ultrasonography did not allow a comprehensive assessment of the complete visceral anatomy, organ sharing, or subtle structural abnormalities.

### 3.4. Hematological Analysis Results

#### 3.4.1. Hematological Analysis of the Dam of the Conjoined Twins

The complete blood count (Table 2) revealed values within the reference range for total leukocytes (10.1 × 10^3^/mm^3^), differential count (lymphocytes, monocytes, and neutrophils), erythrocytes (5.55 × 10^6^/mm^3^), and hemoglobin (15.5 g/dL). A slightly elevated hematocrit (54.2%) was identified. The platelet count (159 × 10^3^/mm^3^) was below the reference range (250–850 × 10^3^/mm^3^), indicating mild thrombocytopenia without clinical manifestations. The erythrocyte indices showed an elevated mean corpuscular volume (MCV; 97.7 fL), increased mean corpuscular hemoglobin (MCH; 27.9 pg), and decreased mean corpuscular hemoglobin concentration (MCHC; 28.5 g/dL), a pattern consistent with mild hypochromic macrocytosis.

#### 3.4.2. Serum Biochemical Analysis of the Dam

The serum biochemical profile (Table 3) showed values within the reference range for all evaluated parameters, including the liver enzymes alanine aminotransferase (ALT; 27.6 U/L), aspartate aminotransferase (AST; 49.1 U/L), gamma-glutamyl transferase (GGT; 3.7 U/L), and alkaline phosphatase (ALP; 35.0 U/L); blood glucose (101.6 mg/dL); and renal function markers, including creatinine (0.72 mg/dL) and urea (34.5 mg/dL).

## 4. Discussion

The present report documents a case of cephalothoraco–omphalopagus conjoined twins in Peru breed guinea pigs from the Cajamarca region of Peru. Both twins were male. Only one previous case of male conjoined twins and one case of female conjoined twins have been reported in guinea pigs from this region [15]. In addition, another case involving the same type of malformation has been described in the Apurímac region, located in the southern Peruvian Andes [16]. To date, only four cases of conjoined twins have been documented in Peru (the present report and the three previously published cases), indicating that this congenital anomaly is uncommon in guinea pigs. In contrast, although still rare, this malformation has been more extensively documented in livestock species such as cattle, goats, and pigs, with anatomical descriptions comparable to those observed in the present case [1,5,17,18].

The classification as cephalothoraco–omphalopagus was based on the anatomical location and extent of the fusion. The presence of a single shared skull corresponded to the cephalopagus classification, fusion of the thoracic wall with a common sternum corresponded to thoracopagus, and fusion of the abdominal region extending to the umbilical level corresponded to omphalopagus [9]. The pelvises were independent and normally developed, as were both the thoracic and pelvic limbs. This classification is consistent with previous reports in humans diagnosed by imaging techniques, including ultrasonography and magnetic resonance imaging, thereby supporting the diagnosis in the present case [19,20]. This type of multiple fusion is less common than simple forms of conjunction, occurring in approximately one out of every 58 cases of conjoined twins, or about one in every three million births [20]. Cephalothoraco–omphalopagus is considered incompatible with life because of the severe anatomical abnormalities associated with this pattern of fusion.

The craniofacial malformations observed, including cyclopia, arhinia, and astomia, were consistent with severe disruption of midline development during neural tube formation (neurulation) [21]. Radiographic examination revealed a single malformed skull, a characteristic finding in this type of anomaly, while most of the appendicular skeleton, particularly the two pairs of thoracic limbs and the two pairs of pelvic limbs, was preserved. The ultrasound examination revealed marked diffuse hyperechogenicity of the lung fields. In addition, two hearts and two pairs of kidneys with preserved gross sonographic morphology and without evident major structural abnormalities.

These results resemble cases previously reported in guinea pigs. Tejml et al. [7] described cephalopagus twins sharing a cranium and sternum but with independently developed limbs, two hearts, and two livers, while sharing lungs. Similarly, Astonitas-Goicochea et al. [15] described two cases of craniothoracopagus twins in Cajamarca, each presenting a single cranium and two vertebral columns, with thoracic and ventral fusion extending to the umbilical region (Zea-Gonzales et al. [16]). They also described conjoined cephalothoraco–omphalopagus twins with a single skull, two vertebral columns, and two hearts. In the present case, ultrasound identified two hearts and two pairs of kidneys with preserved gross sonographic morphology.

However, an important limitation of the present case was the absence of complete post-mortem anatomical dissection and histopathological examination. Preservation of the specimen for academic teaching and display was prioritized, and the academic institution did not have an available veterinary histopathology laboratory. Consequently, the degree of organ duplication and the absence of microscopic tissue abnormalities could not be confirmed, and the assessment of the internal organs was based on findings obtained through diagnostic imaging.

The mechanisms responsible for the development of conjoined twins remain uncertain. The two most widely accepted hypotheses are the incomplete fission theory, which proposes incomplete separation of the embryonic disc before the third week of development, and the secondary fusion theory, which suggests that two initially separate monozygotic embryos subsequently fuse because of their close proximity [8,12,22]. The fusion pattern observed in the present case, characterized by a single skull and extensive ventral union, is consistent with the incomplete fission theory.

From both clinical and productive perspectives, the malformations observed were incompatible with life because of the extensive degree of fusion and the absence of essential anatomical structures required for respiration (arhinia) and feeding (astomia), resulting in immediate perinatal death. The dam recovered uneventfully after parturition, and the two normal littermates developed without complications. However, the available examinations did not identify a specific causal or predisposing factor. The detailed documentation of this case expands the current knowledge of embryological variability in guinea pigs and provides a valuable reference for veterinary education and research in developmental pathology.

## 5. Conclusions

In conclusion, the present case was diagnosed as cephalothoraco–omphalopagus conjoined twins in Peru breed guinea pigs, characterized by a single skull, cyclopia, arhinia, and astomia, with separate pelvises and eight externally developed limbs. Ultrasonic examination identified two hearts and two pairs of kidneys; however, the absence of post-mortem and histopathological examination precluded a complete assessment.

The malformations were incompatible with extrauterine life and did not compromise the health of either the dam or the normal littermates. Because no specific causal or predisposing factor was identified, preventive recommendations cannot be derived from the present case.

## Figures and Tables

**Table 1:** Zoometric measurements of cephalothoraco–omphalopagus conjoined twins in guinea pigs.

Measurements (cm)	Guinea Pig 1	Guinea Pig 2
Head Length (LC)	3.5	3.5
Head Width (AC)	2.1	2.1
Head Circumference (PC)	-	-
Neck Circumference (PCu)	12.32	12.32
Thoracic Circumference (PT)	-	-
Abdominal girth (PA)	12.0	12.3
Hip girth (PCa)	9.3	8.8
Animal length (LA)	13.8	14.2
Body length (LCu)	11.0	12.0
Height at the withers (ACr)	3.8	3.7
Forelimb length (LMa)	7.0	7.5
Hindlimb length (LMp)	8.3	8.7
Cannon bone girth (PC)	3.6	3.8

**Table 2:** Complete blood count of the dam of cojoined twins.

Analysis	Result	Unit/Reference Range
White blood cells (WBC)	10.1	10^3^/mm^3^ (6.0–17.0)
Lymphocytes	5.8	10^3^/mm^3^ (2.5–10.0)
Monocytes	0.7	10^3^/mm^3^ (0.0–1.0)
Neutrophils	3.6	10^3^/mm^3^ (1.5–6.5)
Red blood cells (RBC)	5.55	10^6^/mm^3^ (4.5–7.5)
Hemoglobin	15.5	g/dL (12.0–16.0)
Hematocrit	54.2	% (37.0–48.0)
Platelets	159	10^3^/mm^3^ (250–850)
Mean corpuscular volume (MCV)	97.7	fL (65–80)
Mean corpuscular hemoglobin (MCH)	27.9	pg (20–26)
Mean corpuscular hemoglobin concentration (MCHC)	28.5	g/dL (30–36)

**Table 3:** Serum biochemical profile of the dam of the conjoined twins.

Analysis	Result	Unit/Reference Range
Alanine aminotransferase (ALT)	27.6	U/L (25–60)
Aspartate aminotransferase (AST)	49.1	U/L (35–110)
Gamma-glutamyl transferase (GGT)	3.7	U/L (0.0–10.0)
Alkaline phosphatase (ALP)	35.0	U/L (30–120)
Glucose	101.6	mg/dL (90–140)
Creatinine	0.72	mg/dL (0.4–1.0)
Urea	34.5	mg/dL (20–60)

## Data Availability

The original contributions presented in this study are included in the article.
